# Supplementary material for: Prediction of 5-year progression-free survival in advanced nasopharyngeal carcinoma with pretreatment PET/CT using multi-modality deep learning-based radiomics
Source: Front Oncol. 2022 Jul 29;12:899351. doi: 10.3389/fonc.2022.899351 (PMC9372795; doi:10.3389/fonc.2022.899351)
Supplement: Supplementary file 1 [file DataSheet_1.docx]

Supplementary Material

**Patients and PET/CT Image Data**

Individual treatment protocol was approved by the Nasopharyngeal Carcinoma multidisciplinary team in our institution after the consultation. All patients received Intensity-Modulated Radiation Therapy (IMRT) for a cumulative dose of 66 Gy (2.2 Gy/fraction/day) in 30 fractions for T1 and T2 disease or 70.4 Gy (2.2 Gy/fraction/day) in 32 fractions for T3 and T4 lesion. According to the tumor stage and other clinical characteristics, concomitant chemotherapy or targeted therapy was also performed. Induction chemotherapy was consisted of docetaxel 75 mg/m^2^ on day 1, cisplatin 75 mg/m^2^ on day 1, and 5-Fu 500 mg/m^2^/d continuously on day 1-5. With respect to concurrent chemoradiotherapy (CCRT), cisplatin 40 mg/m^2^ was used weekly during radiation. As for adjuvant chemotherapy, cisplatin 40 mg/m^2^ on day 1-3, and docetaxel 75 mg/m^2^ on day 4 after radiation. Cetuximab was used as a targeted drug with an initial dose of 400 mg/m^2^ followed by 250 mg/m^2^ weekly for the duration of radiotherapy.

^18^F-FDG was produced automatically by cyclotron (Siemens CTI RDS Eclips ST, Knoxville, Tennessee, USA) using Explora FDG_4_ module in our center. The radiochemical purity of ^18^F-FDG was over 95%. Patients were requested to fast 4-6 h to maintain the venous blood glucose levels under 10 mmol/L before scanning. Each patient got injected with 7.4 MBq/kg ^18^F-FDG, then kept lying comfortably in a quiet, dimly lit room for approximately 1 h prior to scanning. Images were obtained on a Siemens biograph 16HR PET/CT scanner (Knoxville, Tennessee, USA). PET/CT data acquisition procedure was as follows: CT scanning was first performed, from the proximal thighs to head, with 120 kV, 80 ~ 250 mA, pitch 3.6, rotation time 0.5, slice thickness: 5.0 mm. Immediately after CT scanning, a PET emission scan that covered the identical transverse field of view was obtained. Acquisition time was 2 ~ 3 min per table position. PET images were reconstructed iteratively using an ordered-subset expectation maximization iterative reconstruction (OSEM) by applying CT images for attenuation correction. The reconstruction parameters were as follows: iterations: 4, subsets: 8, pixel size: 4.0 × 4.0 mm, zoom: 1.0, FWHM: 6.0 mm, and slice thickness: 5.0 mm. Fusion images were reviewed and manipulated on a multimodality computer platform (Syngo, Siemens, Knoxville, Tennessee, USA).

For quantitative analysis, maximum and mean of standardized uptake value (SUV) normalized to body weight were manually computed by drawing a region of interest (ROI). Meanwhile, metabolic tumor volume (MTV) was recorded at the absolute SUV threshold of 2.5. Total lesion glucose (TLG) was calculated according to the formula: TLG = SUV_mean_ × MTV.

**Supplementary Table S1 |** Cox proportional hazard regression analysis for PFS on the internal and external cohorts.

| Characteristics | Internal cohort | | | | | | | External cohort | | | | |
| --- | --- | --- | --- | --- | --- | --- | --- | --- | --- | --- | --- | --- |
|  | Univariate | |  | Multivariate | |  | Univariate | | |  | Multivariate | |
|  | HR (95% CI) | *P* value |  | HR (95% CI) | *P* value |  | HR (95% CI) | | *P* value |  | HR (95% CI) | *P* value |
| Age | 1.02 (1.00-1.05) | 0.066 |  | 1.02 (1.00-1.04) | 0.096 |  | 1.03 (1.00-1.06) | | 0.087 |  | 1.03 (0.99-1.06) | 0.111 |
| Gender |  |  |  |  |  |  |  | |  |  |  | / |
| Male | Reference | / |  | / | / |  | Reference | | / |  | / | / |
| Female | 0.71 (0.34-1.46) | 0.352 |  | / | / |  | 0.85 (0.41-1.75) | | 0.651 |  | / | / |
| EBV antibody |  | 0.199 |  |  | / |  |  | | 0.468 |  |  | / |
| Negative | Reference | / |  | / | / |  | Reference | | / |  | / | / |
| Positive | 0.50 (0.23-1.07) | 0.073 |  | / | / |  | 0.54 (0.19-1.54) | | 0.250 |  | / | / |
| Unknown | 0.65 (0.30-1.40) | 0.275 |  | / | / |  | 0.50 (0.16-1.55) | | 0.231 |  | / | / |
| Histology, WHO Type ^a^ |  | 0.965 |  |  | / |  |  | | / |  |  | / |
| I | Reference | / |  | / | / |  | / | | / |  | / | / |
| II | 1.09 (0.14-8.35) | 0.937 |  | / | / |  | Reference | | / |  | / | / |
| III | 0.99 (0.14-7.25) | 0.994 |  | / | / |  | 3.32 (0.46-24.07) | | 0.236 |  | / | / |
| BMI | 1.06 (0.97-1.15) | 0.224 |  | / | / |  | 1.04 (0.96-1.13) | | 0.327 |  | / | / |
| T stage |  | 0.539 |  |  | / |  |  | | 0.724 |  |  | / |
| T1 | Reference | / |  | / | / |  | Reference | | / |  | / | / |
| T2 | 0.68 (0.22-2.06) | 0.492 |  | / | / |  | 0.62 (0.20-1.96) | | 0.418 |  | / | / |
| T3 | 1.15 (0.58-2.26) | 0.689 |  | / | / |  | 1.14 (0.57-2.25) | | 0.712 |  | / | / |
| T4 | 1.55 (0.67-3.59) | 0.303 |  | / | / |  | 0 | | 0.979 |  | / | / |
| N stage |  | 0.757 |  |  | / |  |  | | 0.358 |  |  | / |
| N0 | Reference | / |  | / | / |  | Reference | | / |  | / | / |
| N1 | 1.06 (0.29-3.84) | 0.932 |  | / | / |  | 2.11 (0.28-16.16) | | 0.471 |  | / | / |
| N2 | 0.90 (0.27-2.97) | 0.858 |  | / | / |  | 1.84 (0.25-13.73) | | 0.551 |  | / | / |
| N3 | 1.34 (0.37-4.88) | 0.655 |  | / | / |  | 3.20 (0.42-24.38) | | 0.261 |  | / | / |
| TNM stage |  |  |  |  |  |  |  | |  |  |  |  |
| III | Reference | / |  | Reference | / |  | Reference | | / |  | Reference | / |
| IVa | 1.80 (1.01-3.21) | 0.047 |  | 1.74 (0.95-3.17) | 0.072 |  | 1.53 (0.82-2.85) | | 0.184 |  | 1.43 (0.75-2.72) | 0.273 |
| PET Parameters |  |  |  |  |  |  |  | |  |  |  |  |
| SUV_max_ | 0.98 (0.93-1.04) | 0.588 |  | / | / |  | 0.98 (0.94-1.02) | | 0.257 |  | / | / |
| SUV_mean_ | 1.02 (0.78-1.34) | 0.875 |  | / | / |  | 0.92 (0.77-1.10) | | 0.351 |  | / | / |
| MTV | 1.01 (1.00-1.02) | 0.051 |  | 1.01 (0.97-1.06) | 0.557 |  | 1.00 (0.98-1.03) | | 0.682 |  | 1.02 (0.98-1.07) | 0.286 |
| TLG | 1.00 (0.99-1.01) | 0.069 |  | 1.00 (0.99-1.01) | 0.706 |  | 1.00 (0.99-1.01) | | 0.901 |  | 1.00 (0.99-1.00) | 0.519 |

^a^ WHO Type I = keratinizing, WHO Type II = non-keratinizing (differentiated), WHO Type III = non-keratinizing (undifferentiated).

*PFS, progression-free survival; HR, hazard rate; CI, confidence interval; EBV, Epstein–Barr virus; WHO, World Health Organization; BMI, body mass index; SUV, standardized uptake value; MTV, metabolic tumor volume; TLG, total lesion glycolysis.*

**Supplementary Table S2 |** Cox proportional hazard regression analysis for PFS using discrete PET parameters.

| Characteristics | Internal cohort | | | | |  | External cohort | | | | |
| --- | --- | --- | --- | --- | --- | --- | --- | --- | --- | --- | --- |
|  | Univariate | |  | Multivariate | |  | Univariate | |  | Multivariate | |
|  | HR (95% CI) | *P* value |  | HR (95% CI) | *P* value |  | HR (95% CI) | *P* value |  | HR (95% CI) | *P* value |
| Age | 1.02 (1.00-1.05) | 0.066 |  | 1.02 (1.00-1.04) | 0.127 |  | 1.03 (1.00-1.06) | 0.087 |  | 1.03 (1.00-1.06) | 0.107 |
| TNM stage |  |  |  |  |  |  |  |  |  |  |  |
| III | Reference | / |  | Reference | / |  | Reference | / |  | Reference | / |
| IVa | 1.80 (1.01-3.21) | 0.047 |  | 1.51 (0.82-2.78) | 0.182 |  | 1.53 (0.82-2.85) | 0.184 |  | 1.40 (0.74-2.64) | 0.303 |
| PET Parameters |  |  |  |  |  |  |  |  |  |  |  |
| SUV_max_ _cutoff ^a^ | 1.68 (0.71-3.94) | 0.237 |  | / | / |  | 0.86 (0.46-1.60) | 0.627 |  | / | / |
| SUV_mean_ _cutoff | 1.68 (0.71-3.95) | 0.236 |  | / | / |  | 1.15 (0.58-2.25) | 0.693 |  | / | / |
| MTV_cutoff | 2.50 (1.40-4.44) | 0.002 |  | 1.35 (0.30-6.00) | 0.694 |  | 1.22 (0.52-2.88) | 0.650 |  | 0.95 (0.13-7.13) | 0.963 |
| TLG _cutoff | 2.49 (1.40-4.42) | 0.002 |  | 1.58 (0.36-6.98) | 0.544 |  | 1.27 (0.57-2.83) | 0.559 |  | 1.56 (0.24-10.15) | 0.644 |

^a^ The cutoff values for SUV_max_, SUV_mean_, MTV and TLG were 17.71 g/ml, 6.24 g/ml, 39.80 ml and 198.68 g, respectively.

*PFS, progression-free survival; HR, hazard rate; CI, confidence interval; SUV, standardized uptake value; MTV, metabolic tumor volume; TLG, total lesion glycolysis.*
